# Protocols and their effects for medical device-related pressure injury prevention among critically ill patients: a systematic review

**DOI:** 10.1186/s12912-024-02080-y

**Published:** 2024-06-17

**Authors:** Haeyoung Lee, Seunghye Choi

**Affiliations:** 1 Red Cross College of Nursing, Chung-Ang University, 84 Heukseok-ro, Dongjak-Gu, Seoul, 06974 South Korea; 2 College of Nursing, Gachon University, 191, Hambangmoe-ro, Yeonsu-gu, Incheon, 21936 South Korea

**Keywords:** Medical device, Intensive care unit, Prevention, Critically ill patient, Pressure injury

## Abstract

**Background:**

A pressure injury refers to localized damage to the skin and/or tissue due to prolonged pressure, and it has recently been defined to include pressure injuries related to medical devices. Medical device-related pressure injuries occur in various sites and are difficult to detect. Even if it is detected, medical devices are essential to life for critically ill patients. Thus, it is difficult to remove or change the position of the medical device; therefore, prevention is essential. This study aims to integrate the literature on medical device-related pressure injury prevention protocols among critically ill patients.

**Methods:**

The literature inclusion criteria were (1) critically ill patients, (2) device-related pressure injury interventions, (3) randomized controlled trials and quasi-experimental designs, and (4) written in Korean or English. The literature search and selection were performed following the Cochrane Handbook for Systematic Reviews of Interventions with the support of the PRISMA Guidelines.

**Results:**

Twelve articles were finally selected. The incidence of medical device-related pressure injury decreased from 8.1–96.7% before intervention to 0.3–53.3% after intervention, respectively. Medical device-related pressure injury prevention was effective in reducing medical device-related pressure injury incidence when applied to patients of all ages, from neonates to adults, in a variety of intensive care units. Medical device-related pressure injury prevention strategies include nurse education, assessment, documentation, and interventions (hygiene, repositioning, emergent therapy such as protective dressing or designed equipment reducing pressure) of pressure injury. Pressure injury dressings primarily included hydrocolloid foam dressings, but transparent hydrocolloid formulations also effectively reduced medical device-related pressure injury incidence rates.

**Conclusions:**

In the future, it is necessary to increase the level of evidence by applying specialized medical device-related pressure injury prevention methods for different medical devices and areas of pressure injuries, and verifying their effectiveness.

**Trial registration:**

The review protocol was registered (PROSPERO registration number: CRD42022346450).

**Supplementary Information:**

The online version contains supplementary material available at 10.1186/s12912-024-02080-y.

## Background

A pressure injury (PI) comprises localized damage to the skin and/or underlying soft tissue usually over a bony prominence as a result of prolonged pressure or pressure in combination with shear [1, 2]; it has recently been defined to include PIs related to medical devices [2]. PI is associated with ineffective tissue perfusion or excessive deformation of the tissue [3]. Sustained external pressures above a threshold cause prolonged ischemia, and reperfusion injury, which occurs when the blood supply is restored after a period of ischemia. This is considered an additional cause of tissue damage that causes PI. Moreover, the shear and friction may be factors affecting local capillary beds, which could be contributing to tissue hypoxia [4]. Tissue damage can occur not only with short periods of high pressure, but also with prolonged periods of low pressure [3]. In particular, medical device-related pressure injuries (MDRPIs) do not occur at bony protrusions like typical PIs, but at various sites such as skin and mucous membranes where medical devices are applied, making it difficult to detect and accurately assess the depth of PIs [2].

The incidence of PI is an indicator of the quality of care and hospitals are applying practices to prevent PI; however, its incidence in intensive care units (ICUs) ranges from 21 to 35%, higher than 3 to 14% observed in general wards [5]. PI often occurs in people with impaired mobility or sensation [4]. Especially, critically ill patients often have uncontrollable external and internal factors that make it difficult to avoid the development of PI despite the implementation of PI preventive care [6]. Previous studies have identified approximately 43 risk factors for PI in critically ill patients, which can be categorized into intrinsic factors such as patient characteristics, length of stay, comorbidities, and hypotension; medical devices such as prolonged mechanical ventilation; and vasopressor agents [7]. In a previous study that examined 2,203 cardiovascular ICU patients over a three-year period, the incidence of PI in the ICU was 24.4%, with 79.5% of the cases comprising stage 2 or higher PI at initial diagnosis [8]. The occurrence of PI is related to a prolonged treatment period, which increases the cost of hospitalization [9] and the incidence of mortality and complications if not treated appropriately [10]. Therefore, it is urgent to establish protocols for the prevention and early detection of MDRPI as well as general PI [11].

The current MDRPI prevention protocol is based on international evidence-based PI guidelines [12], but its use in clinical practice is limited due to the wide variety of medical devices associated with MDRPI and the difficulty of easily changing their location due to the nature of medical devices [13]. Therefore, a systematic review of ICU MDRPI protocols is needed to provide an empirical basis for the development of PI prevention algorithms applicable in the ICU. This study aims to integrate the literature on the protocols for medical device-related pressure injury prevention among critically ill patients of all ages.

## Methods

### Study design

This study was a systematic review that investigated the interventions for MDRPI prevention among critically ill patients. The review protocol was registered (PROSPERO registration number: CRD42022346450). The literature search and selection were performed in accordance with the Cochrane Handbook for Systematic Reviews of Interventions [14] and the Preferred Reporting Items of Systematic Reviews and Meta-Analyses (PRISMA-P) checklist for systematic reviews [15].

### Inclusion and exclusion criteria

The inclusion criteria are delineated using PICO-SD (Population, Intervention, Comparison, Outcome, Study Design) framework as follows: (1) P: Critically ill patients with MDRPI, (2) I: PI prevention protocol, (3) C and (4) O: Not specified during the literature search, and (5) SD: Randomized controlled trials (RCTs) and quasi-experimental designs. Furthermore, literature written in both Korean and English languages was encompassed in the study selection process. The exclusion criteria were as follows: (1) the patient already had a medical device-related injury prior to ICU admission, and (2) the study involved animals.

### Data search and collection process

#### Data search strategy

We searched databases based on the Core Standard Ideal (COSI) model theory [16], and selected PubMed (https://pubmed.ncbi.nlm.nih.gov/), EMBASE (https://www.embase.com/), Cochrane Library (https://www.cochranelibrary.com/), and CINAHL (https://search.ebscohost.com/), which are mainly used in the medical field. Three information retrieval experts carried out a methodologically sound search for the literature.

Regarding search terms, we used MeSH terms in PubMed and Cochrane Library, and Emtree terms in EMBASE. We also added related natural language and converted it into search expressions by combining Boolean operators (AND, OR, NOT) between search terms. For the high sensitivity, we searched the literature using a combination of terms corresponding to P and I without specifying the terms of C and O.

As for P (critically ill patients with MDRPI), MeSH terms, including “Critical Care,” “Critical Illness,” “Intensive Care Units,” “Hospitalization,” “Life Support Care,” “Equipment and Supplies,” and “Pressure Ulcer”; and Emtree terms, including “intensive care,” “critical illness,” “intensive care unit,” “intensive care medicine,” “hospitalization,” “long term care,” “medical device,” “decubitus,” and “medical device related pressure ulcer,” as well as natural languages, were selected as search terms.

Regarding I (PI prevention protocol), MeSH terms, including “prevention and control,” “Clinical Protocols,” “Patient Care Bundles,” and “Algorithms”; and Emtree terms, including “prevention,” “clinical protocol,” “care bundle,” and “algorithm,” as well as natural languages, were selected as search terms. All studies published after 1975 were included in the initial search. The search was conducted between August 5 and August 21, 2022. After deduplication, 2,121 articles were retrieved, and the final search expression is presented in Table S1.

#### Screening process and data extraction

**Fig. 1:**
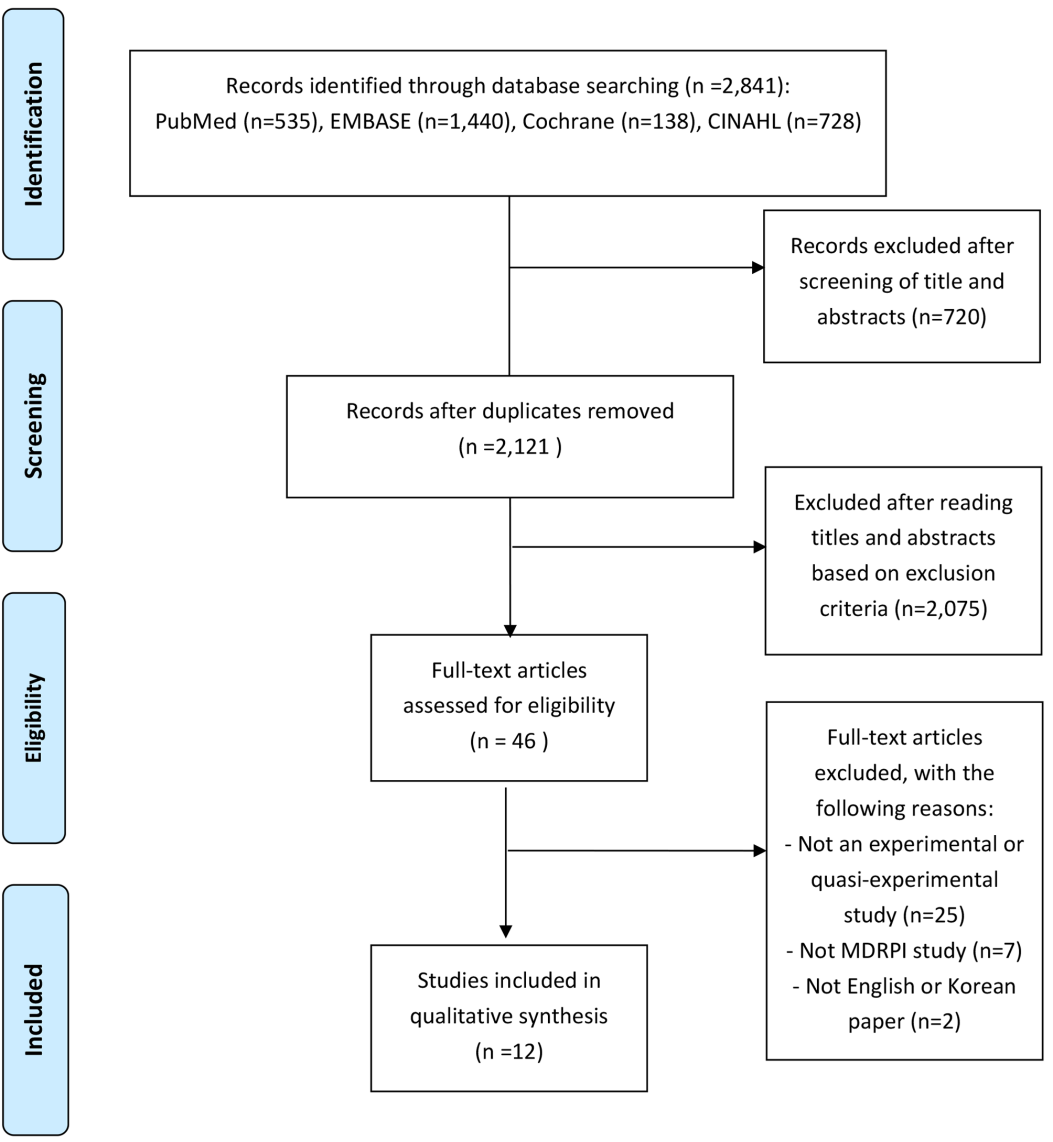
PRISMA flow chart for the literature selection process

The 2,121 retrieved articles were organized in Excel and ENDNOTE, and two researchers independently reviewed the literature. In the first step, the titles and abstracts were reviewed to select articles to be included in the study. In the second step, the full texts were reviewed to select articles for inclusion in the study, and any disagreements were resolved through discussion. Twelve articles were finally selected (Fig. 1), and their authors, study titles, journal names, years, volumes (issues), ICU, patient disease, MDRPI areas, MDRPI staging tool, type of medical device causing injury, sample size (experimental and control group), type of intervention, MDRPI prevention instruments, intervention time/session/frequency, primary outcome, and secondary outcome (if applied) were noted.

#### Quality appraisal and synthesis of results

The revised Cochrane risk of bias tool for randomized trials (ROB 2) was used to assess RCT quality, and the Risk of Bias in Non-randomized Studies of Interventions (ROBINS-I) version 2.0 was used to assess non-RCT quality [14]. Two authors independently assessed the full text of each article and then reached a consensus on the conclusions. The final 12 articles were then integrated through a qualitative synthesis method.

## Results

### Study selection

**Fig. 2:**
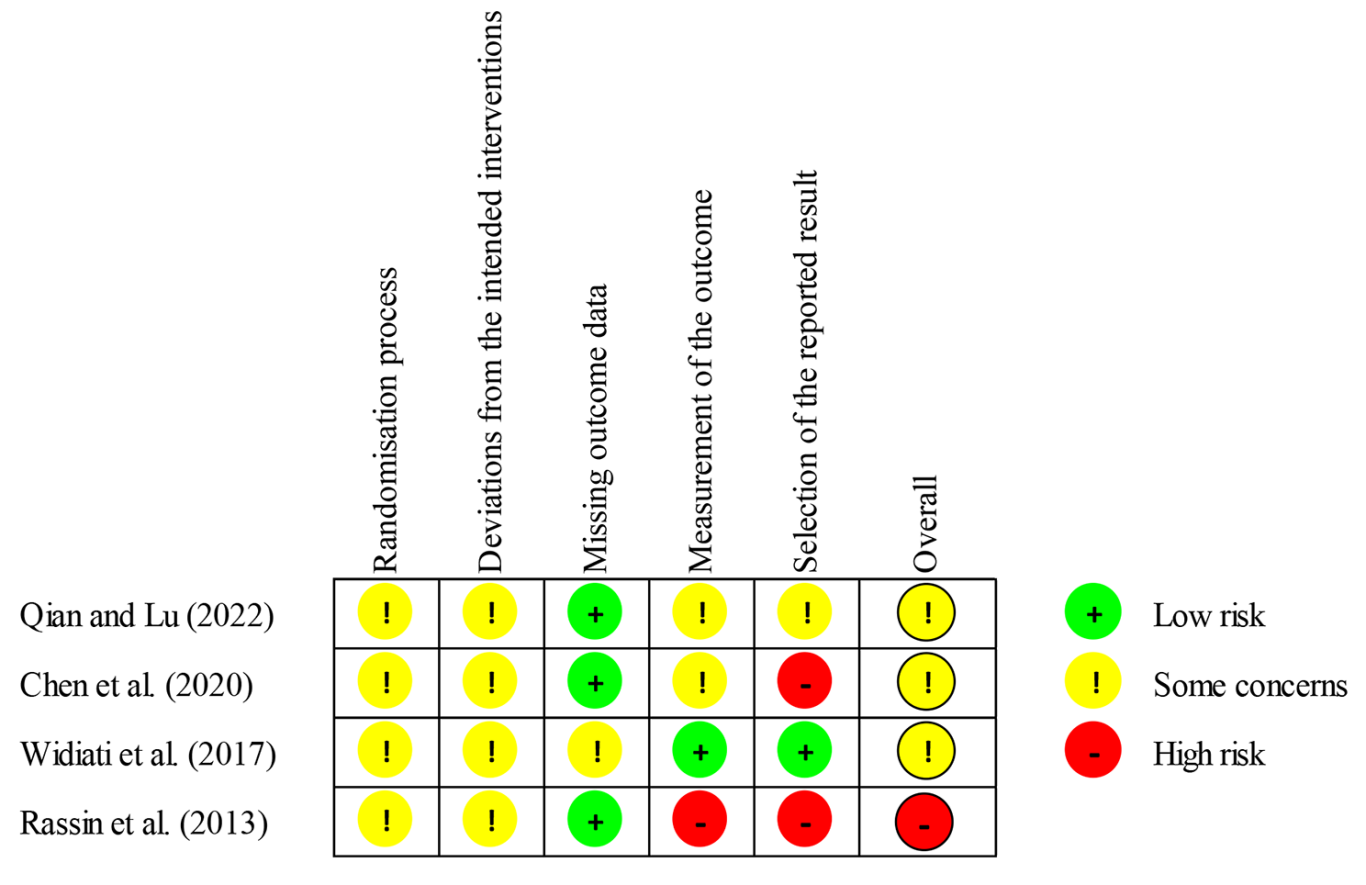
Risk of bias in randomized trials (*N* = 4)

A total of 12 articles were selected based on the inclusion criteria. According to the search strategy, 2,841 articles were retrieved, 535 from PubMed, 1,440 from EMBASE, 138 from the Cochrane Library, and 728 from CINAHL. After excluding duplicates, 2,121 articles were reviewed. Two researchers reviewed the titles and abstracts and excluded 2,075 articles based on the exclusion criteria. We reviewed the full text of 46 articles, out of which we excluded 34 articles for the following reasons: not an experimental or quasi-experimental study (25 studies), not a study on MDRPI (7 studies), and not in English or Korean (2 studies) (Fig. 1). The assessments of the risk of bias in the selected articles are presented in Table 1; Fig. 2.

**Table 1:** Risk of bias in non-randomized studies of intervention (ROBINS-I) (*N* = 8)

Study	Bias due to Confounding	Bias in selection of participants for the study	Bias in classification of intervention	Bias due to deviations from intended intervention	Bias due to missing data	Bias in measurement of outcome	Bias in selection of the reported result	Overall bias
Arundel et al. (2021) [17]	Serious	NI	Serious	Critical	NI	Critical	Critical	Critical
Tayyib et al. (2021) [18]	Moderate	Low	Low	Low	Moderate	Moderate	Moderate	Moderate
Mietzsch et al. (2019) [19]	Serious	NI	Serious	Low	NI	Low	Low	Moderate
Boesch et al. (2012) [20]	Serious	Serious	Serious	Low	NI	Low	Low	Serious
Weng (2008) [21]	Moderate	Low	Low	Moderate	Moderate	Moderate	Low	Moderate
Krzyzewski et al. (2022) [22]	Serious	Low	Moderate	Moderate	NI	Moderate	Low	Serious
Zakaria et al. (2018) [23]	Low	Serious	Serious	Low	Low	Low	Low	Serious
Coyer et al. (2015) [24]	Serious	Low	Low	Low	NI	Low	NI	Serious

NI; no information

### Characteristics of included studies and participants

Of the 12 studies, 8 were non-RCTs (Table 1) and 4 were RCTs (Fig. 2). Five studies included adult ICU patients, five included pediatric patients, one included both adults and pediatric patients, and one did not report patient age. Articles were published in 2008 (*n* = 1), 2012 (*n* = 1), 2013 (*n* = 1), 2015 (*n* = 1), 2017 (*n* = 1), 2018 (*n* = 1), 2019 (*n* = 1), 2020 (*n* = 1), 2021 (*n* = 2), and 2022 (*n* = 2) (Table 2).

### Characteristics of MDRPI

Among the 12 papers, the types of medical devices and sites where PIs occurred varied. One article did not specify a medical device and included all medical devices, with others including respiratory system-related masks or tubes (*n* = 6), endo-tracheal (ETT) and nasogastric tubes (NGT) (*n* = 3), continuous electroencephalographic (cEEG) electrodes (*n* = 1), and a foley catheter-related PI in a male patient (*n* = 1) (Table 2). Therefore, as the site of the MDRPI, the face (nose, nostrils, lips, and cheeks) (*n* = 11) and medical device insertion sites (below or above stoma, under twill ties, ETT or NGT insertion site, SpO_2_ contacts, and urinary meatus) were often assessed (Table 2). As for MDRPI staging tools, most of the papers used the pressure ulcer staging system checklist (PUSS) developed by the NPIAP (National Pressure Injury Advisory Panel) [29, 30] (*n* = 5) (Table 2). Other studies used a standardized assessment tool designed by the researchers (*n* = 5) [31, 32] and other tools (*n* = 2) (Table 2).

### Characteristics and effects of MDRPI prevention interventions for critically ill patients

For MDRPI prevention interventions, seven articles used care bundles or guidelines that included assessment, documentation, and performance frequency for MDRPI prevention; two articles used protective dressings at the site of medical device application; two articles used specially designed equipment; and one article designed a nursing intervention that included cleaning, catheter placement, cushioning dressings, and immobilization methods such a special positioning of the device to distribute skin pressure (Table 3). The shortest interval between MDRPI assessment was 30 min [21], and in most papers, the interval was 3 to 4 h (*n* = 3) (Table 3). Interventions most often included an interprofessional team approach (*n* = 5), followed by those provided by nurses (*n* = 4) (Table 3).

### Primary and secondary outcomes of studies in this systematic review

The primary outcome assessed in studies included in this systematic review was the change in the incidence or occurrence rate of MDRPI among critically ill patients after the application of interventions. The study found that in most cases (*n* = 9), MDRPI was significantly lower post-intervention compared to pre-intervention (Table 4). Specifically, this study reported reductions in MDRPI rates from 13.4 to 0.89% [18], 8.5–3.5% [19], 8.1–0.3% [20], 96.7–53.3% or 40% [21], 90–32.1% [23], 77.8–13.1% [23], 30.4–18.1% [24], 12.1–0.86% [25], 72.6–43.3% [26], 28.6–24.1% [28], and 67.3–38.6% [28] before and after intervention, respectively (Table 4). Regarding secondary outcomes, besides the incidence rate, notable findings included a decrease in abscesses and infections related to the PI from 22.2% pre-intervention to 0% post-intervention (*n* = 1) [19]. The duration until a PI occurred was also significantly different between the intervention and control groups (*n* = 2) [20, 21], and the median survival times of the nasal skin integrity were significantly higher in the experimental group than in the control group (*n* = 1) [26]. Additionally, the comfort level of patients was significantly higher in the intervention group than in the control group, and the degree of tracheal tube displacement was significantly less in the intervention group than in the control group (*n* = 1) [25] (Table 4).

### Quality assessment

The quality of RCT studies (*n* = 4) was assessed using ROB 2 (Fig. 2), and the risk of bias in Non-RCT studies (*n* = 8) was assessed using ROBINS-I (Table 1). According to the ROB 2 evaluation, the overall biases were categorized as high risk (*n* = 1) and some concerns (*n* = 3) (Fig. 2). Conversely, the overall biases using ROBINS-I were classified as critical (*n* = 1), serious (*n* = 4), and moderate (*n* = 3) (Table 1).

**Table 2:** General characteristics of included studies (*N* = 12)

Author (year)	Country	Study design	Institution	Setting	Age of participants
Arundel et al. (2021) [17]	USA	Quality improvement	A Magnet-designated, 182-bed community hospital in the mid-Atlantic region	12-bed critical care unit (patient age not reported)	Not reported
Tayyib et al. (2021) [18]	Saudi Arabia	A pilot prospective, single-arm, open-label clinical design	A Saudi Arabian tertiary hospital	CCU (Two adult CCUs and one pediatric unit)	Mean (SD, IQR) Adults: 67y (22.49, 18-102y) Pediatric: 27.57mo (26.3, 1-120mo)
Mietzsch et al. (2019) [19]	USA	Quality improvement	Riley Hospital for Children	NICU (neonates)	Neonates, not reported specific age
Boesch et al. (2012) [20]	USA	Quality improvement	A 490-bed academic quaternary-care, free-standing children’s hospital	18-bed ventilator unit (children)	Median (IQR) 2y, 8mo (13mo to 9y)
Weng (2008) [21]	Taiwan	Quasi-experimental method	A medical center in northern Taiwan	MICU, CCU (adults diagnosed with respiratory failure)	Mean (SD) Tegaderm group: 75.2 (13.3) Tegasorb group: 79.1 (10.5) Control group: 75.0 (12.2)
Krzyzewski et al. (2022) [22]	USA	Quality improvement project	Johns Hopkins all children’s hospital	97-bed level IV NICU (neonates)	Infants, not reported specific age
Zakaria et al. (2018) [23]	Egypt	Prospective, quasi-experimental research (before and after design)	Not reported	Adult ICU	Mean (SD, range) 47.42y (10.44, 41-50y)
Coyer et al. (2015) [24]	Australia	Quasi-experimental design (before and after design)	A 36-bed general adult ICU in an Australian metropolitan tertiary referral hospital, the Royal Brisbane and Women’s Hospital	Adult ICU admits general medical, surgical, and trauma adult patients	Median (IQR) 59.3y (45-70y)
Qian and Lu (2022) [25]	China	Non-double blinded RCT	Not reported	The department of surgery and critical care medicine (age ≥ 18 years old)	Adults 18 years and older, not reported specific age
Chen et al. (2020) [26]	China	RCT	A tertiary medical center in southern China	PICU	Median (IQR) Experimental group: 16mo (5.25–45.75) Control group: 12mo (3–36)
Widiati et al. (2017) [27]	Indonesia	RCT with a crossover design	Not reported	PICU	Mean (SD) Neonatal age: 17.98d(13.67) Pediatric age: 7.5y(6.8)
Rassin et al. (2013) [28]	Israel	Non-double blinded RCT	Not reported	Respiratory ICU (male adult)	Mean (SD) Research group: 63.7y (15.7) Control group: 60y (19.9)

Author (year)	Characteristics of Subjects	Site of MDRPI	MDRPI staging tool	Injury causing medical devices
Arundel et al. (2021) [17]	Not reported	On the nasal bridge	Debrief tool for MDRPI related to CPAP/BiPAP mask	CPAP/BiPAP Masks
Tayyib et al. (2021) [18]	Trauma (2.69%), illness related to medical problem (69.5%), postsurgery (8.07%), and sepsis/infectious disease (15.69%)	Nares	standard physical assessment tool designed by the researcher	Any medical device that causes damage to skin, tissue, or mucous membranes.
Mietzsch et al. (2019) [19]	All neonates monitored with cEEG	cEEG electrode–related skin injury consistent with contact dermatitis was most frequently seen in areas of the face and electrocardiographic electrodes.	PUSS developed by the NPUAP	Electrode for cEEG monitoring
Boesch et al. (2012) [20]	Ventilation-dependent children admitted for acute illness, surgical procedures, or diagnostic testing	Below the tracheostomy stoma: *n* = 16 (73%) Above flanges: *n* = 3 (14%) Above stoma: *n* = 2 (9%) Under twill ties: *n* = 1 (4%)	PUSS developed by the NPUAP	Tracheostomy tube
Weng (2008) [21]	Respiratory failure, non-invasive ventilation patients	Facial skin lesions	Pressure ulcers were classified into four grades.	NIV face mask
Krzyzewski et al. (2022) [22]	Breathing premature infants in the neonatal ICU being supported by NIV	Nasal septum: 6 (38%) Nasal bridge: 9 (56%) Side of nose: 1(6%)	PUSS developed by the NPUAP (Stage I PIs were excluded)	NIV device
Zakaria et al. (2018) [23]	Critically ill adult male and female patients	ETT and or NGT insertion site	PUSS developed by the NPUAP	ETT and NGT
Coyer et al. (2015) [24]	Neurology/respiratory/Trauma/Sepsis/Cardiovascular/Renal and metabolic/Abdominal disorders	Lip and nares	A standardized skin assessment tool based on assessment via physical examination and common sites for development of pressure injuries. It was used to standardize clinical examination among the research nurses. Pressure injury were divided into skin and mucosal injuries.	ETT and NGT
Qian and Lu (2022) [25]	Patients with mechanical ventilation trough orotracheal intubation	The skin around the mouth, cheeks, and neck	The staging of oral ulcers Stage I pressure ulcers: flaky or streak-like bruising on the skin around the lips and gums Stage II pressure ulcers: the skin around the lips and gums is purple-red, with blisters and superficial mucosal ulceration Stage III pressure ulcer: a superficial ulcer stage, with full-thickness skin destruction, which can penetrate deep into the subcutaneous tissue and deep tissue	Orotracheal intubation
Chen et al. (2020) [26]	Pediatric patients received invasive mechanical ventilation vial nasotracheal tubes	Nasal skin pressure ulcers	PUSS developed by the NPUAP	NGT
Widiati et al. (2017) [27]	Not reported	ETT OGT, NGT, SpO_2_ probe contact area	Take a picture to verify	ETT (13%), OGT (12%), NGT (11%), and SpO_2_ probe (6%)
Rassin et al. (2013) [28]	Male patients (septic shock, respiratory failure, trauma, kidney failure)	Urinary meatus	Not reported	Foley catheter

SD; standard deviation, IQR; Interquartile Range, y; years, mo; months, d; days, RCT; randomized controlled trial, ICU; intensive care unit, NICU; neonatal ICU, MICU; medical ICU, PICU; pediatric ICU; CCU, coronary care unit

cEEG; continuous electroencephalographic, ICU; intensive care unit, ETT; endo-tracheal tube, NGT; nasogastric tube, OGT; orogastric tube, CPAP; continuous positive airway pressure, BiPAP; bilevel positive airway pressure, PUSS; pressure ulcer staging system checklist, NPUAP; National Pressure Ulcer Advisory Panel; NIV; noninvasive ventilation

**Table 3:** Characteristics of studies included in this systematic review (*N* = 12)

Author (year)	Sample size		Experimental intervention			
	Experiment	Control	Type of intervention	Provider	Intervention instruments	Frequency
Arundel et al. (2021) [17]	All patients who visited in 2017	All patients who visited in 2016	Evidence-based guidelines (baseline facial skin assessment and the performance, and correct documentation of every four-hour facial skin assessment while CPAP/BiPAP was in use)	Interprofessional team approach -Nurses were educated to perform a baseline skin assessment of the face, forehead, and the nose bridge. If any abnormalities were found, they were reported by the charging RN and respiratory therapist using the debrief tool. - Skin with a problem is dressed by the wound, ostomy, and continence nurse using a thin foam dressing.	Thin foam dressing (Molnlyke Mepilex Lite, Peachtree Corners, Georgia) Updated non-invasive BiPAP/CPAP mask for pressure redistribution (Philips Respironics AF541, Murrysville, Pennsylvania)	Facial skin assessments at the time of admission and every four hours for the duration of the NIVM therapy
Tayyib et al. (2021) [18]	223 persons (adults 131, pediatric 92, respectively)	None - Arbitrarily set a typical the three-month MDRPI average incidence of 13.5% according to The National Database Nursing Quality Indicators as a baseline	The SKINCARE bundle (prevention strategies for PI development such as nursing clinical assessment and documentation, hygiene measures, repositioning, and emerging therapy for MDRPI prevention for critically ill patients)	RNs (approximately 400 RNs) -Bachelor’s degree in nursing, mandatory advanced critical care training, and relevant clinical experience.	Thin hydrocolloid, single-layer silicone foam, and silicone tape.	Every three hours for a total of 24 h
Mietzsch et al. (2019) [19]	198	106	The monitoring tool kit	Multidisciplinary task force team - Neonatology, neurology, neurophysiology, nursing, and wound care	Use a flexible stick cotton swab, and apply conduction paste (Ten 20, Weaver and Company, Aurora, Colorado) Apply softgel-based electrodes	The wound care nurse assessed the patients for 18 months of the project period. The assessment intervals were not reported.
Boesch et al. (2012) [20]	834	136 patients seen in the 6 months prior to the intervention	TRPU prevention bundle (frequent skin and device assessments, moisture-reducing device interface, and pressure-free device interface.)	All TRPUs were identified by a bedside nurse and, all TRPUs were reported to and staged by a wound-care expert.	Hydrophilic barrier used under the tracheostomy tube flanges and around the stoma. Extended tracheostomy tubes were used.	Once a week
Weng (2008) [21]	Exp. Group I: Tegasorb group 30 Exp. Group II: Tegaderm group 30	30	Protective treatment (covered with tegasorb or tegaderm dressing)	Not reported.	Tegasorb, tegaderm *Tegasorb is easy to observe the skin condition through its transparent structure. **Tegaderm is permeable to water vapor	Every 30-min checking of the skin condition
Krzyzewski et al. (2022) [22]	Post NIV guideline 424 Post SCB 243 Sustainability 321	Pre NIV guideline 290	PI prevention bundle	Multidisciplinary team This included notification of the medical team, consultation with a wound-ostomy nurse practitioner for assessment and staging, and entry of the injury into an internal PI database as well as entry into an electronic hospital safety reporting system when a PI was identified	A thin foam dressing was used as a pressure barrier and placed on the nasal bridge over the hydrocolloid barrier when using a nasal mask	Every three-hour skin assessment Absolute number of stage 2 or worse and deep tissue pressure injuries reported per month for 36 months.
Zakaria et al. (2018) [23]	48	52	ETT: choice of correct size, lark head to tie, avoidance of fixation by adhesive tabbing, placing of a pad, avoidance of tying the ETT fixation tape under the head; the repositioning of the ETT every two hours	Ten highly qualified RNs	Lark head to tie the ETT. Placing of a pad between skin and ETT. The use of a water-soluble lubricant during insertion of NGTs. The wetting of the NGT adhesive tape with warm water before removal	Recently connected with oral ETT and/ or NGT within 48 h from date of insertion at day zero of data collection up to three weeks.
Coyer et al. (2015) [24]	105	102	Skin integrity protocol bundle, the InSPiRE protocol	Specialist intensive care medical practitioners responsible for admission and management, and registered nurses provide all care.	Non-powered pressure-redistribution support surface, a dynamic powered alternating pressure support surface, or another support surface	12 months: daily data were collected on patients from recruitment to discharge form the ICU or death
Qian and Lu (2022) [25]	116	116	Apply a self-designed oral fluid suction device to fix the tracheal intubation	Patients were assessed by uniformly trained nurses.	Oral fluid suction device The main body is used to absorb oral fluid, which is made of multiple layers of gauze wound in a spiral manner. The adjacent gauze layers of each layer are stacked in sequence.	Post-intubation immobilization and observation
Chen et al. (2020) [26]	60	62	Hydrocolloid dressing to protect nasal skin from the beginning of nasotracheal intubation	Physician and nurse	Hydrocolloid dressing	The hydrocolloid dressing was changed daily to assess the nasal skin.
Widiati et al. (2017) [27]	50	50 (cross-over design)	Precautionary treatments based on Kiss and Heiler’s guidelines (Assess the skin with a medical device, and take a picture)	Not reported	Medical treatment based on Kiss and Heiler’s guidelines	Assessment frequency not reported, observed for three days
Rassin et al. (2013) [28]	Phase I 29 Phase II 57	28 55	Phase I: the area around the catheter entry point was washed with soap and water, and the catheter placement was switched to the other thigh, where it was cushioned with a gauze pad and held with adhesive tape. Phase II: Same intervention method, different number of times	Nursing staff	The catheter placement site was cushioned with a gauze pad	Phase I: once every 24 h Phase II: once on each shift, that is, 3 times every 24 h Data collection continued for approximately 18 months

CPAP; continuous positive airway pressure, BiPAP; bilevel positive airway pressure, RN; registered nurse, NIVM; noninvasive ventilation mask, TRPU; tracheostomy related pressure ulcer, NIV; noninvasive ventilation, SCB; skin care bundle, ETT; endo-tracheal tube, NGT; nasogastric tube, ICU; intensive care unit

**Table 4:** Primary and secondary outcomes of studies in this systematic review (*N* = 12)

Author (year)	Control group	Key interventions	Primary outcome	Secondary outcome	Key findings
Arundel et al. (2021) [17]	Standard routine care	ᆞApplying protective dressings ᆞRepositioning	Incidence of CPAP-/ BiPAP-related MDRPI		Only one stage 1 injury was identified, and it resolved quickly with the appropriate assessments and interventions. This showed a 75% reduction in actual injuries with a zero escalation to stage 2 or greater injuries
Tayyib et al. (2021) [18]	Not reported	ᆞApplying protective dressings ᆞCleaning the surface area ᆞChoosing the right size of medical equipment ᆞRepositioning	The development of MDRPI localized injury to the skin and underlying tissue, including mucous membranes, caused by pressure from an external medical device.		MDRPI incidence was 0.89%, a significant decrease from baseline 13.4%.
Mietzsch et al. (2019) [19]	Not reported	ᆞCleaning the surface area	Reduction in the Incidence of PU	Elimination of skin abscesses and infections (electrode- related infections)	Reduced PU incidence from 8.5% (9/106) before intervention to 3.5% (7/198) of monitored patients during the project period. Abscesses and infections related to the PU occurred in 22.2% of patients with PUs before the intervention, and no infections occurred after the intervention.
Boesch et al. (2012) [20]	Standard routine care	ᆞApplying protective dressings	TRPU occurrence rates (new TRPUs per month/number of tracheostomy patients in the unit that month)	TRPU bed days (days associated with a TRPU per month/total number of unit bed days with a tracheostomy tube)	TRPU incidence rates decreased from the baseline period (8.1%) to the intervention period (0.3%). TRPU bed days decreased from the baseline period (12.5%) to the intervention period (0.2%).
Weng (2008) [21]	Standard routine care	ᆞApplying protective dressings	Occurrence of pressure ulcers	Occurrence duration time	The occurrence of pressure ulcers was significantly less in the tegaderm (53.3%) and tegasorb groups (40%) compared to the control group (96.7%). The duration time of pressure ulcer was significantly longer in the tegaderm (2628 ± 1655 min) and tegasorb groups (3272 ± 2566 min) compared to the control group (1111 ± 2169), but there was no significant difference between the experimental groups.
Krzyzewski et al. (2022) [22]	Standard routine care	ᆞApplying protective dressings	The incidence of NIV device-related PI		The mean incidence rate of NIV device-related PI per 1,000 NICU patient days for each phase was as follows: 0.05 (pre-NIV guideline) 0.42 (post-NIV guideline) 0.08 (post SCB) 0.16 (sustainability)
Zakaria et al. (2018) [23]	Standard routine care	ᆞApplying protective dressings ᆞChoosing the right size of medical equipment ᆞRepositioning	Incidence of ETT related PUs incidence of NGT related PUs		The incidence of ETT-related PUs decreased from 90–32.1% The incidence of NGT-related PUs fell from 77.8–13.1%
Coyer et al. (2015) [24]	Standard routine care	ᆞCleaning the surface area ᆞRepositioning ᆞElimination of pressure and friction ᆞProtection against forces of pressure and friction (maintenance of stable skin temperature, optimizing nutritional status, and promotion of mobility)	Cumulative incidence of PIs	PIs develop later in their ICU stay. PIs per patient. Frequency of care for PIs.	The cumulative incidence of PIs was significantly different between the intervention group (18.1%) and the control group (30.4%). The intervention group had 19 patients with 24 PIs and the control group had 31 patients with 64 PIs. The intervention group had (17/102) only 1 PI and fewer skin injuries (4/105) compared to the control group. The number of skin integrity assessments was not significantly different between the experimental and control groups.
Qian and Lu (2022) [25]	Standard routine care; Used the traditional method, placing ordinary disposable tooth pads, and then using 3 M tape to fix the tracheal intubation	ᆞDesigning of a new suction device	The incidence of oral mucosa and lip pressure ulcers	Patient comfort assessment (VAS) Tracheal tube displacement	I. Oral and lip pressure: experimental group- mild (1/116), moderate (0/116) vs. control group- mild (14/116), moderate (2/116) The incidence of oral cavity mucous membrane PU: experimental group (1/116) vs. control group (10/116) The incidence of mild stage oral and lip pressure were significantly decreased in the experimental group (*p* < 0.001). II. Comfort level: experimental group-comfortable (100/116) vs. control group-comfortable (50/116) III. Tracheal tube displacement: experimental group (1/116) vs. control group (16/116)
Chen et al. (2020) [26]	Standard routine care; The current care procedure (without hydrocolloid dressing) unless PIs occurred	ᆞApplying protective dressings	NTT-related PIs	The median survival times of the nasal skin integrity	Forty-five participants had NTT-related PIs in the control group, whereas 26 patients had NTT-related PIs in the experimental group (72.6% vs. 43.3%; absolute difference, 29.3%, 95% CI, 12.5–46%; *p* = 0.001). The median survival times of the nasal skin integrity were 95.5 h in the control group and 219.5 h in the experimental group (*p* < 0.001).
Widiati et al. (2017) [27]	Standard routine care; Received PI prevention treatment following the hospital routines	ᆞApplying protective dressings ᆞRepositioning	The number of PI incidents		The number of PI incidents was 57.1%, 33.3%, and 42.9% for the intervention group on days 1, 2, and 3, respectively, and 42.9%, 66.7%, and 57.1% for the control group, respectively.
Rassin et al. (2013) [28]	Once every 24 h, the area around the catheter entry point was washed with soap and water.	ᆞApplying protective dressings ᆞCleaning the surface area ᆞRepositioning	Occurrence of PUs		Phase I: Research group (24.1%) vs. control group (28.6%) Phase II: Research group (38.6%) vs. control group (67.3%; *p* = 0.002)

CPAP; continuous positive airway pressure, BiPAP; bilevel positive airway pressure, ETT; endo-tracheal tube, NGT; nasogastric tube, PI; pressure injury, PU; pressure ulcer, NTT; nasotracheal tube, TRPU; tracheostomy-related pressure ulcer, NICU; neonatal ICU, NIV; noninvasive ventilation, SCB; skin care bundle; CI, confidence interval

## Discussion

PIs in critically ill patients adversely affect patient outcomes [33]. Since 2016, when the National Pressure Injury Advisory Panel revised the PI staging system to include damage caused by medical devices [34], medical devices have been recognized as a significant risk factor for PI [30]. Against this backdrop, this systematic review aimed to investigate the literature on the protocols for MDRPI prevention among critically ill patients of all ages. Fourteen studies met our inclusion criteria, only adult (*n* = 5), from neonate to pediatric (*n* = 2), not reported or mixed age (*n* = 2). The majority (62.5%) in the non-randomized studies (*n* = 8) were assessed as serious to critical bias, and just 37.5% were classified as moderate bias. In the RCT studies (*n* = 4), the risk of biases were some concerns (*n* = 3), and high risk (*n* = 1). Our results highlight the need for the development of evidence-based RCT studies.

The pre-intervention MDRPI incidence varied from 8.1 to 96.7%, which includes the stage I PI (intact skin with non-blanchable redness of a localized area) [23, 30]; this range was higher than that of 0.9–41.2% in a previous study of critically ill patients [35]. It is believed the incidence of MDRPIs was significantly reduced in the studies using MDRPI prevention strategies, including careful assessment, accurate documentation, protective dressings to prevent MDRPIs, selection of appropriately sized medical devices, and proper immobilization to prevent tissue damage.

The ICUs implementing prevention strategies in this study were from various departments, and the age of the population was almost equally divided between adults and pediatric patients. Even with MDRPI occurring in the same site, protective dressing options for adults and neonatal or pediatric might be different for the following reasons: Nostrils of neonatal and pediatric are so small that only thin dressings are applicable, and a dressing without adhesion poses a risk of entering into the nasal cavity [26]. In this study, thin foam dressing was used in the neonatal study [22], but multiple layers of gauze were used in the adult study [25]. These results cannot be generalized, but when applying the MDRPI prevention strategies, confirming whether it suits the participants’ age will be crucial.

MDRPI prevention strategies in most studies focused on preventing PIs caused by the specific medical devices studied, most of which were respiratory-related [17, 20–26]. In a study that included all medical devices that cause skin, tissue, and mucosal damage without specifying a particular medical device, PIs occurred 100% of the time in nares [18]. This is probably because respiratory support devices are used for the longest time among critically ill patients. It is often difficult for critically ill patients to avoid the development of PI, despite the implementation of PI prevention nursing care, due to uncontrollable external and internal factors [6]. Interventions were performed to prevent PI from electrode-related injury and its secondary infections in premature infants [19]. In premature infants, it may take four weeks or more for the skin barrier to form [36]; thus, care should be taken with continuous monitoring due to a potential for PI at the electrode attachment site.

This systematic review identified seven articles that employed evidence-based care bundles or guidelines for MDRPI prevention interventions [17–20, 22, 24, 27]. These studies predominantly adopted a multidisciplinary approach, incorporating nurse education, PI assessment, PI documentation, and various PI interventions as part of their strategies. Through an interprofessional team approach, respiratory therapists trained nurses on how to properly release and reattach the continuous positive airway pressure/ bilevel positive airway pressure (CPAP/BiPAP) mask using the straps, while the charge nurse periodically assessed the skin and immediately recorded any redness or breakdown of skin using a prescribed form; the wound, ostomy, and continence nurse performed pressure redistribution using thin foam [17]. In another paper, the SKINCARE bundle [18] was used to help nurses assess, document, ensure hygiene, reposition, and provide emerging therapies for MDRPI prevention (e.g., protective dressings for high-risk areas and selecting the right size of device for the individual) [18, 20]. These evidence-based interventions appeared to be effective, as MDRPI incidence was lower post-intervention than pre-intervention in studies using evidence-based care bundles or guidelines, except one [22]. As even nurses can have difficulty handling medical devices [19], and physician consent is often required to resize or reposition medical devices to fit the patient [37], a multidisciplinary approach to MDRPI prevention strategies in ICU patients would be more effective. Moreover, in the case of MDRPI in the ICU, 79.5% of the cases involved stage 2 or higher PIs at the first detection [8]; thus, early detection through routine assessment is likely to be crucial for patient prognosis.

Interventions included cleaning the surface area [18, 19, 24, 28], choosing the right size of medical equipment [18, 23], applying protective dressings [17, 18, 20–23, 26–28], repositioning [17, 18, 23, 24, 27, 28], elimination of pressure and friction [24], protection against forces of pressure and friction (maintenance of stable skin temperature, optimizing nutritional status, and promotion of mobility) [24], and the designing of a new suction device [25]. Dressing types primarily included hydrocolloid foam dressings [17, 18, 20, 26], but transparent hydrocolloid formulations were also used in some cases to allow observation of the skin beneath the device [21, 22]. In the case of MDRPI, it is not easy to observe before removing the medical device; thus, it is believed that the appropriate use of transparent hydrocolloid dressings may be beneficial. A study comparing Tegasorb and Tegaderm found a reduction in the incidence of PIs compared to a no-dressing control group, with no significant difference between dressing types [19]. These results, however, were based on facial skin lesions in adult patients [21]; thus, replication studies with different subjects and body part injuries are required.

Most of the MDRPI assessment tools in this study used the PUSS checklist developed by the NPUAP [19, 20, 22, 23, 26, 29, 30]. However, as various MDRPIs can present with lesions at different sites, modified or investigator-standardized staging tools were more commonly used [17, 18, 21, 24, 25, 27, 28]. The most critical factor in MDRPI prevention involves the accurate measurement of the extent of skin and underlying tissues injury. However, not only do MDRPIs develop more rapidly than non-MDRPIs [38], but it is often difficult to accurately assess the skin underneath a medical device [12]; thus, it is essential to specialize the staging tool according to the type of medical device and site of occurrence.

### Limitations

Despite the significance of this study, there are a few limitations to be acknowledged. First, the MDRPIs included in this study used different medical devices, various patients, protocols, and providers. Therefore, the results cannot be generalized, and it is necessary to conduct repeated RCT studies on MDRPI protocols applicable to specific participants. Secondly, a meta-analysis is more appropriate when a set of studies investigates identical or closely related relationships and is derived from similar research designs. In the present study, we have included studies with heterogeneity in terms of study quality, subjects, outcome variables, and intervention methods. Therefore, we have opted for a qualitative synthesis method instead of conducting a meta-analysis. Thirdly, another significant limitation lies in the absence of skin tone information for the participants in the studies. Consequently, we could not evaluate how diverse skin tones might influence MDRPI or impact the effectiveness of MDRPI prevention strategies. Finally, many studies were conducted as quality improvement projects, and the quality assessment showed that the papers were not of high quality, suggesting a need for higher quality evidence.

## Conclusions

MDRPI prevention was found to be associated with a decreased incidence of MDRPI in patients of different ages in a variety of ICUs. MDRPI prevention strategies included nurse education/PI assessment/PI documentation/PI interventions (hygiene, repositioning, emergent therapy). PI dressings primarily included hydrocolloid foam dressings, but transparent hydrocolloid formulations were also effective in reducing the incidence of MDRPI. Depending on the age group, the utilization of different PI dressings may be necessary. Therefore, a specialized interprofessional team approach is needed depending on the type of medical device and site of the occurrence. Since it is difficult to detect MDRPI early, it is necessary to educate and support nurses to develop competency in MDRPI assessment and care while establishing a systematic nursing record system that can support appropriate documentation, including images, to build a better healthcare system.

### Electronic supplementary material

Below is the link to the electronic supplementary material.

Supplementary Material 1

## Data Availability

The data that support the findings of this study are available from the corresponding author upon reasonable request.

## Abbreviations

MDRPI: medical device-related pressure injury
PI: pressure injury
ICU: intensive care unit
RCT: randomized controlled trial
PRISMA: preferred reporting items of systematic reviews and meta-analyses
PICO: population, intervention, control, and outcomes
COSI: core standard ideal
ROB: risk of bias
ROBINS-I: risk of bias in non-randomized studies of interventions
ETT: endo-tracheal tube
NGT: nasogastric tube
PUSS: pressure ulcer staging system
NPUAP: national pressure ulcer advisory panel
PU: pressure ulcer
CPAP: continuous positive airway pressure
BiPAP: bilevel positive airway pressure
