# Supplementary material for: Protocols and their effects for medical device-related pressure injury prevention among critically ill patients: a systematic review
Source: BMC Nurs. 2024 Jun 17;23:403. doi: 10.1186/s12912-024-02080-y (PMC11181566; doi:10.1186/s12912-024-02080-y)
Supplement: Supplementary file 1 — Supplementary Material 1 [file 12912_2024_2080_MOESM1_ESM.docx]

Table S1. Final search formula for the systematic review

1)Pubmed

| #1 | "Critical Care"[Mesh] | 64,689 |
| --- | --- | --- |
| #2 | "Critical Care"[TW] OR "Care, Critical"[TW] OR "Intensive Care"[TW] OR "Care, Intensive"[TW] OR "Surgical Intensive Care"[TW] OR "Care, Surgical Intensive"[TW] OR "Intensive Care, Surgical"[TW] OR "intensive therapy"[TW] OR "therapy, intensive"[TW] | 262,052 |
| #3 | "Critical Illness"[Mesh] | 36,589 |
| #4 | "Critical Illness"[TW] OR "Critical Illnesses"[TW] OR "Illness, Critical"[TW] OR "Illnesses, Critical"[TW] OR "Critically Ill"[TW] OR "serious illness"[TW] OR "seriously ill"[TW] | 80,345 |
| #5 | "Intensive Care Units"[Mesh] | 101,090 |
| #6 | "Intensive Care Units"[TW] OR "Intensive Care Unit"[TW] OR "Unit, Intensive Care"[TW] OR "ICU Intensive Care Units"[TW] OR "close attention unit"[TW] OR "combined medical and surgical ICU"[TW] OR "combined surgical and medical ICU"[TW] OR "critical care unit"[TW] OR "general ICU"[TW] OR "GICU"[TW] OR "GICUs"[TW] OR "ICU's"[TW] OR "intensive care department"[TW] OR "intensive therapy unit"[TW] OR "intensive treatment unit"[TW] OR "medical-surgery ICU"[TW] OR "medical/surgical ICU"[TW] OR "medical/surgical ICUs"[TW] OR "medico-surgical ICU"[TW] OR "mixed medical and surgical ICU"[TW] OR "mixed surgical and medical ICU"[TW] OR "respiratory care unit"[TW] OR "respiratory care units"[TW] OR "special care unit"[TW] OR "surgery/medical ICU"[TW] OR "surgical-medical ICUs"[TW] OR "surgical/medical ICU"[TW] OR "ICU"[TW] OR "SICU"[TW] OR "ITU"[TW] OR "CCU"[TW] OR "CTU"[TW] OR "HDU"[TW] | 206,152 |
| #7 | "intensive care medicine"[TW] OR "critical care medicine"[TW] | 6,939 |
| #8 | "Hospitalization"[Mesh] | 281,793 |
| #9 | "Hospitalization"[TW] OR "Hospitalizations"[TW] OR "hospital stay"[TW] OR "short stay hospitalization"[TW] | 342,992 |
| #10 | "Life Support Care"[Mesh] | 9,065 |
| #11 | "Life Support Care"[TW] OR "Care, Life Support"[TW] OR "Prolongation of Life"[TW] OR "Life Prolongation"[TW] OR "Extraordinary Treatment"[TW] OR "Extraordinary Treatments"[TW] OR "Treatment, Extraordinary"[TW] OR "Treatments, Extraordinary"[TW] OR "chronic treatment"[TW] OR "long term medical care"[TW] OR "long term therapy"[TW] OR "long term treatment"[TW] OR "long-term care"[TW] OR "medical care, long term"[TW] OR "treatment, long term"[TW] OR "coronary care"[TW] OR "cardiothoracic unit"[TW] | 102,606 |
| #12 | "critical patients"[TW] OR "critically ill patients"[TW] | 36,775 |
| **#13** | **#1 OR #2 OR #3 OR #4 OR #5 OR #6 OR #7 OR #8 OR #9 OR #10 OR #11 OR #12** | **814,177** |
| #14 | "Equipment and Supplies"[Mesh] | 1,600,343 |
| #15 | "Equipment and Supplies"[TW] OR "Supplies and Equipment"[TW] OR "Apparatus and Instruments"[TW] OR "Instruments and Apparatus"[TW] OR "Supplies"[TW] OR "Inventories"[TW] OR "Inventory"[TW] OR "Medical Devices"[TW] OR "Medical Device"[TW] OR "Device, Medical"[TW] OR "Devices, Medical"[TW] OR "Devices"[TW] OR "Device"[TW] OR "Equipment"[TW] OR "bioinstrumentation"[TW] OR "biological instrumentation"[TW] OR "biomedical device"[TW] OR "biomedical device (physical object)"[TW] OR "biomedical equipment"[TW] OR "biomedical instrumentation"[TW] OR "clinical device"[TW] OR "clinical equipment"[TW] OR "durable medical equipment"[TW] OR "instrumentation, biological"[TW] OR "instrumentation, medical"[TW] OR "medical apparatus"[TW] OR "medical apparatus, equipment and supplies"[TW] OR "medical equipment"[TW] OR "medical instrument"[TW] OR "medical instrumentation"[TW] OR "medical supplies"[TW] OR "device-related"[TW] OR "medical device related"[TW] OR "appliances"[TW] OR "support surfaces"[TW] | 1,029,688 |
| **#16** | **#14 OR #15** | **2,270,795** |
| #17 | "Pressure Ulcer"[Mesh] | 13,275 |
| #18 | "Pressure Ulcer"[TW] OR "Pressure Ulcers"[TW] OR "Ulcer, Pressure"[TW] OR "Ulcers, Pressure"[TW] OR "Bedsore"[TW] OR "Bedsores"[TW] OR "Pressure Sore"[TW] OR "Pressure Sores"[TW] OR "Sore, Pressure"[TW] OR "Sores, Pressure"[TW] OR "Bed Sores"[TW] OR "Bed Sore"[TW] OR "Sore, Bed"[TW] OR "Sores, Bed"[TW] OR "Decubitus Ulcer"[TW] OR "Decubitus Ulcers"[TW] OR "Ulcer, Decubitus"[TW] OR "Ulcers, Decubitus"[TW] OR "decubital ulcer"[TW] OR "decubital ulcus"[TW] OR "decubitus ulceration"[TW] OR "decubitus ulcus"[TW] OR "decubus ulcer"[TW] OR "pressure injury"[TW] OR "ulcus decubitus"[TW] OR "pressure damage"[TW] | 18,510 |
| **#19** | **#17 OR #18** | **18,510** |
| #20 | "medical device related pressure ulcer"[TW] OR "device related pressure injury"[TW] OR "device related pressure ulcer"[TW] OR "MDRPI"[TW] OR "MDRPU"[TW] OR "medical device related pressure injury"[TW] OR "Medical Device-Related Pressure Injuries"[TW] | 93 |
| **#21** | **(#16 AND #19) OR #20** | **5,500** |
| **#22** | **#13 AND #21** | **811** |
| #23 | "prevention and control"[Subheading] | 1,423,360 |
| #24 | "prophylaxis"[TW] OR "preventive therapy"[TW] OR "prevention and control"[TW] OR "preventive measures"[TW] OR "prevention"[TW] OR "control"[TW] | 4,652,533 |
| #25 | "Clinical Protocols"[Mesh] | 185,797 |
| #26 | "Clinical Protocols"[TW] OR "Protocol, Clinical"[TW] OR "Protocols, Clinical"[TW] OR "Clinical Protocol"[TW] OR "Treatment Protocols"[TW] OR "Protocols, Treatment"[TW] OR "Treatment Protocol"[TW] OR "Clinical Research Protocol"[TW] OR "Research Protocols, Clinical"[TW] OR "Protocols, Clinical Research"[TW] OR "Research Protocol, Clinical"[TW] OR "Clinical Research Protocols"[TW] OR "Protocol, Clinical Research"[TW] OR "protocol"[TW] OR "protocols"[TW] | 697,478 |
| #27 | "Patient Care Bundles"[Mesh] | 1,167 |
| #28 | "Patient Care Bundles"[TW] OR "Bundle, Patient Care"[TW] OR "Bundles, Patient Care"[TW] OR "Care Bundle, Patient"[TW] OR "Care Bundles, Patient"[TW] OR "Patient Care Bundle"[TW] OR "Care Bundles"[TW] OR "Bundle, Care"[TW] OR "Bundles, Care"[TW] OR "Care Bundle"[TW] OR "healthcare bundle"[TW] OR "Bundle"[TW] OR "Bundles"[TW] | 74,288 |
| #29 | "Algorithms"[Mesh] | 403,617 |
| #30 | "Algorithms"[TW] OR "Algorithm"[TW] OR "algorhythm"[TW] OR "algorism"[TW] | 479,372 |
| **#31** | **#23 OR #24 OR #25 OR #26 OR #27 OR #28 OR #29 OR #30** | **5,757,623** |
| **#32** | **#22 AND #31** | **535** |

2)Embase

| #1 | "intensive care"/exp | 836,008 |
| --- | --- | --- |
| #2 | "Critical Care":ti,ab,kw,de OR "Care, Critical":ti,ab,kw,de OR "Intensive Care":ti,ab,kw,de OR "Care, Intensive":ti,ab,kw,de OR "Surgical Intensive Care":ti,ab,kw,de OR "Care, Surgical Intensive":ti,ab,kw,de OR "Intensive Care, Surgical":ti,ab,kw,de OR "intensive therapy":ti,ab,kw,de OR "therapy, intensive":ti,ab,kw,de | 459,538 |
| #3 | "critical illness"/exp | 33,645 |
| #4 | "Critical Illness":ti,ab,kw,de OR "Critical Illnesses":ti,ab,kw,de OR "Illness, Critical":ti,ab,kw,de OR "Illnesses, Critical":ti,ab,kw,de OR "Critically Ill":ti,ab,kw,de OR "serious illness":ti,ab,kw,de OR "seriously ill":ti,ab,kw,de | 126,287 |
| #5 | "intensive care unit"/exp | 258,459 |
| #6 | "Intensive Care Units":ti,ab,kw,de OR "Intensive Care Unit":ti,ab,kw,de OR "Unit, Intensive Care":ti,ab,kw,de OR "ICU Intensive Care Units":ti,ab,kw,de OR "close attention unit":ti,ab,kw,de OR "combined medical and surgical ICU":ti,ab,kw,de OR "combined surgical and medical ICU":ti,ab,kw,de OR "critical care unit":ti,ab,kw,de OR "general ICU":ti,ab,kw,de OR "GICU":ti,ab,kw,de OR "GICUs":ti,ab,kw,de OR "ICU^s":ti,ab,kw,de OR "intensive care department":ti,ab,kw,de OR "intensive therapy unit":ti,ab,kw,de OR "intensive treatment unit":ti,ab,kw,de OR "medical-surgery ICU":ti,ab,kw,de OR "medical/surgical ICU":ti,ab,kw,de OR "medical/surgical ICUs":ti,ab,kw,de OR "medico-surgical ICU":ti,ab,kw,de OR "mixed medical and surgical ICU":ti,ab,kw,de OR "mixed surgical and medical ICU":ti,ab,kw,de OR "respiratory care unit":ti,ab,kw,de OR "respiratory care units":ti,ab,kw,de OR "special care unit":ti,ab,kw,de OR "surgery/medical ICU":ti,ab,kw,de OR "surgical-medical ICUs":ti,ab,kw,de OR "surgical/medical ICU":ti,ab,kw,de OR "ICU":ti,ab,kw,de OR "SICU":ti,ab,kw,de OR "ITU":ti,ab,kw,de OR "CCU":ti,ab,kw,de OR "CTU":ti,ab,kw,de OR "HDU":ti,ab,kw,de | 371,252 |
| #7 | "intensive care medicine":ti,ab,kw,de OR "critical care medicine":ti,ab,kw,de | 15,096 |
| #8 | "hospitalization"/exp | 474,660 |
| #9 | "Hospitalization":ti,ab,kw,de OR "Hospitalizations":ti,ab,kw,de OR "hospital stay":ti,ab,kw,de OR "short stay hospitalization":ti,ab,kw,de | 635,656 |
| #10 | "long term care"/exp | 2,130,735 |
| #11 | "Life Support Care":ti,ab,kw,de OR "Care, Life Support":ti,ab,kw,de OR "Prolongation of Life":ti,ab,kw,de OR "Life Prolongation":ti,ab,kw,de OR "Extraordinary Treatment":ti,ab,kw,de OR "Extraordinary Treatments":ti,ab,kw,de OR "Treatment, Extraordinary":ti,ab,kw,de OR "Treatments, Extraordinary":ti,ab,kw,de OR "chronic treatment":ti,ab,kw,de OR "long term medical care":ti,ab,kw,de OR "long term therapy":ti,ab,kw,de OR "long term treatment":ti,ab,kw,de OR "long-term care":ti,ab,kw,de OR "medical care, long term":ti,ab,kw,de OR "treatment, long term":ti,ab,kw,de OR "coronary care":ti,ab,kw,de OR "cardiothoracic unit":ti,ab,kw,de | 214,038 |
| #12 | "critical patients":ti,ab,kw,de OR "critically ill patients":ti,ab,kw,de | 54,870 |
| **#13** | **#1 OR #2 OR #3 OR #4 OR #5 OR #6 OR #7 OR #8 OR #9 OR #10 OR #11 OR #12** | **3,645,182** |
| #14 | "medical device"/exp | 3,532,900 |
| #15 | "Equipment and Supplies":ti,ab,kw,de OR "Supplies and Equipment":ti,ab,kw,de OR "Apparatus and Instruments":ti,ab,kw,de OR "Instruments and Apparatus":ti,ab,kw,de OR "Supplies":ti,ab,kw,de OR "Inventories":ti,ab,kw,de OR "Inventory":ti,ab,kw,de OR "Medical Devices":ti,ab,kw,de OR "Medical Device":ti,ab,kw,de OR "Device, Medical":ti,ab,kw,de OR "Devices, Medical":ti,ab,kw,de OR "Devices":ti,ab,kw,de OR "Device":ti,ab,kw,de OR "Equipment":ti,ab,kw,de OR "bioinstrumentation":ti,ab,kw,de OR "biological instrumentation":ti,ab,kw,de OR "biomedical device":ti,ab,kw,de OR "biomedical device (physical object)":ti,ab,kw,de OR "biomedical equipment":ti,ab,kw,de OR "biomedical instrumentation":ti,ab,kw,de OR "clinical device":ti,ab,kw,de OR "clinical equipment":ti,ab,kw,de OR "durable medical equipment":ti,ab,kw,de OR "instrumentation, biological":ti,ab,kw,de OR "instrumentation, medical":ti,ab,kw,de OR "medical apparatus":ti,ab,kw,de OR "medical apparatus, equipment and supplies":ti,ab,kw,de OR "medical equipment":ti,ab,kw,de OR "medical instrument":ti,ab,kw,de OR "medical instrumentation":ti,ab,kw,de OR "medical supplies":ti,ab,kw,de OR "device-related":ti,ab,kw,de OR "medical device related":ti,ab,kw,de OR "appliances":ti,ab,kw,de OR "support surfaces":ti,ab,kw,de | 1,698,243 |
| **#16** | **#14 OR #15** | **4,422,160** |
| #17 | "decubitus"/exp | 25,531 |
| #18 | "Pressure Ulcer":ti,ab,kw,de OR "Pressure Ulcers":ti,ab,kw,de OR "Ulcer, Pressure":ti,ab,kw,de OR "Ulcers, Pressure":ti,ab,kw,de OR "Bedsore":ti,ab,kw,de OR "Bedsores":ti,ab,kw,de OR "Pressure Sore":ti,ab,kw,de OR "Pressure Sores":ti,ab,kw,de OR "Sore, Pressure":ti,ab,kw,de OR "Sores, Pressure":ti,ab,kw,de OR "Bed Sores":ti,ab,kw,de OR "Bed Sore":ti,ab,kw,de OR "Sore, Bed":ti,ab,kw,de OR "Sores, Bed":ti,ab,kw,de OR "Decubitus Ulcer":ti,ab,kw,de OR "Decubitus Ulcers":ti,ab,kw,de OR "Ulcer, Decubitus":ti,ab,kw,de OR "Ulcers, Decubitus":ti,ab,kw,de OR "decubital ulcer":ti,ab,kw,de OR "decubital ulcus":ti,ab,kw,de OR "decubitus ulceration":ti,ab,kw,de OR "decubitus ulcus":ti,ab,kw,de OR "decubus ulcer":ti,ab,kw,de OR "pressure injury":ti,ab,kw,de OR "ulcus decubitus":ti,ab,kw,de OR "pressure damage":ti,ab,kw,de | 19,161 |
| **#19** | **#17 OR #18** | **29,183** |
| #20 | "medical device related pressure ulcer"/exp OR "medical device related pressure ulcer":ti,ab,kw,de OR "device related pressure injury":ti,ab,kw,de OR "device related pressure ulcer":ti,ab,kw,de OR "MDRPI":ti,ab,kw,de OR "MDRPU":ti,ab,kw,de OR "medical device related pressure injury":ti,ab,kw,de OR "Medical Device-Related Pressure Injuries":ti,ab,kw,de | 134 |
| **#21** | **(#16 AND #19) OR #20** | **9,936** |
| **#22** | **#13 AND #21** | **3,383** |
| #23 | "prevention"/exp | 1,670,095 |
| #24 | "prevention and control":ti,ab,kw,de OR "prophylaxis":ti,ab,kw,de OR "preventive therapy":ti,ab,kw,de OR "prevention and control":ti,ab,kw,de OR "preventive measures":ti,ab,kw,de OR "prevention":ti,ab,kw,de OR "control":ti,ab,kw,de | 5,571,232 |
| #25 | "clinical protocol"/exp | 114,232 |
| #26 | "Clinical Protocols":ti,ab,kw,de OR "Protocol, Clinical":ti,ab,kw,de OR "Protocols, Clinical":ti,ab,kw,de OR "Clinical Protocol":ti,ab,kw,de OR "Treatment Protocols":ti,ab,kw,de OR "Protocols, Treatment":ti,ab,kw,de OR "Treatment Protocol":ti,ab,kw,de OR "Clinical Research Protocol":ti,ab,kw,de OR "Research Protocols, Clinical":ti,ab,kw,de OR "Protocols, Clinical Research":ti,ab,kw,de OR "Research Protocol, Clinical":ti,ab,kw,de OR "Clinical Research Protocols":ti,ab,kw,de OR "Protocol, Clinical Research":ti,ab,kw,de OR "protocol":ti,ab,kw,de OR "protocols":ti,ab,kw,de | 849,508 |
| #27 | "care bundle"/exp | 1,757 |
| #28 | "Patient Care Bundles":ti,ab,kw,de OR "Bundle, Patient Care":ti,ab,kw,de OR "Bundles, Patient Care":ti,ab,kw,de OR "Care Bundle, Patient":ti,ab,kw,de OR "Care Bundles, Patient":ti,ab,kw,de OR "Patient Care Bundle":ti,ab,kw,de OR "Care Bundles":ti,ab,kw,de OR "Bundle, Care":ti,ab,kw,de OR "Bundles, Care":ti,ab,kw,de OR "Care Bundle":ti,ab,kw,de OR "healthcare bundle":ti,ab,kw,de OR "Bundle":ti,ab,kw,de OR "Bundles":ti,ab,kw,de | 108,615 |
| #29 | "algorithm"/exp | 506,730 |
| #30 | "Algorithms":ti,ab,kw,de OR "Algorithm":ti,ab,kw,de OR "algorhythm":ti,ab,kw,de OR "algorism":ti,ab,kw,de | 531,551 |
| **#31** | **#23 OR #24 OR #25 OR #26 OR #27 OR #28 OR #29 OR #30** | **7,631,792** |
| **#32** | **#22 AND #31** | **1,440** |

3)Cochrane Library

| #1 | [mh "Critical Care"] | 2,232 |
| --- | --- | --- |
| #2 | "Critical Care":ti,ab,kw OR "Care, Critical":ti,ab,kw OR "Intensive Care":ti,ab,kw OR "Care, Intensive":ti,ab,kw OR "Surgical Intensive Care":ti,ab,kw OR "Care, Surgical Intensive":ti,ab,kw OR "Intensive Care, Surgical":ti,ab,kw OR "intensive therapy":ti,ab,kw OR "therapy, intensive":ti,ab,kw | 29,497 |
| #3 | [mh "Critical Illness"] | 2,639 |
| #4 | "Critical Illness":ti,ab,kw OR "Critical Illnesses":ti,ab,kw OR "Illness, Critical":ti,ab,kw OR "Illnesses, Critical":ti,ab,kw OR "Critically Ill":ti,ab,kw OR "serious illness":ti,ab,kw OR "seriously ill":ti,ab,kw | 9,812 |
| #5 | [mh "Intensive Care Units"] | 4,120 |
| #6 | "Intensive Care Units":ti,ab,kw OR "Intensive Care Unit":ti,ab,kw OR "Unit, Intensive Care":ti,ab,kw OR "ICU Intensive Care Units":ti,ab,kw OR "close attention unit":ti,ab,kw OR "combined medical and surgical ICU":ti,ab,kw OR "combined surgical and medical ICU":ti,ab,kw OR "critical care unit":ti,ab,kw OR "general ICU":ti,ab,kw OR "GICU":ti,ab,kw OR "GICUs":ti,ab,kw OR "ICU's":ti,ab,kw OR "intensive care department":ti,ab,kw OR "intensive therapy unit":ti,ab,kw OR "intensive treatment unit":ti,ab,kw OR "medical-surgery ICU":ti,ab,kw OR "medical/surgical ICU":ti,ab,kw OR "medical/surgical ICUs":ti,ab,kw OR "medico-surgical ICU":ti,ab,kw OR "mixed medical and surgical ICU":ti,ab,kw OR "mixed surgical and medical ICU":ti,ab,kw OR "respiratory care unit":ti,ab,kw OR "respiratory care units":ti,ab,kw OR "special care unit":ti,ab,kw OR "surgery/medical ICU":ti,ab,kw OR "surgical-medical ICUs":ti,ab,kw OR "surgical/medical ICU":ti,ab,kw OR "ICU":ti,ab,kw OR "SICU":ti,ab,kw OR "ITU":ti,ab,kw OR "CCU":ti,ab,kw OR "CTU":ti,ab,kw OR "HDU":ti,ab,kw | 31,476 |
| #7 | "intensive care medicine":ti,ab,kw OR "critical care medicine":ti,ab,kw | 581 |
| #8 | [mh "Hospitalization"] | 15,401 |
| #9 | "Hospitalization":ti,ab,kw OR "Hospitalizations":ti,ab,kw OR "hospital stay":ti,ab,kw OR "short stay hospitalization":ti,ab,kw | 62,915 |
| #10 | [mh "Life Support Care"] | 161 |
| #11 | "Life Support Care":ti,ab,kw OR "Care, Life Support":ti,ab,kw OR "Prolongation of Life":ti,ab,kw OR "Life Prolongation":ti,ab,kw OR "Extraordinary Treatment":ti,ab,kw OR "Extraordinary Treatments":ti,ab,kw OR "Treatment, Extraordinary":ti,ab,kw OR "Treatments, Extraordinary":ti,ab,kw OR "chronic treatment":ti,ab,kw OR "long term medical care":ti,ab,kw OR "long term therapy":ti,ab,kw OR "long term treatment":ti,ab,kw OR "long-term care":ti,ab,kw OR "medical care, long term":ti,ab,kw OR "treatment, long term":ti,ab,kw OR "coronary care":ti,ab,kw OR "cardiothoracic unit":ti,ab,kw | 14,816 |
| #12 | "critical patients":ti,ab,kw OR "critically ill patients":ti,ab,kw | 5,538 |
| **#13** | **{OR #1-#12}** | **115,077** |
| #14 | [mh "Equipment and Supplies"] | 53,231 |
| #15 | "Equipment and Supplies":ti,ab,kw OR "Supplies and Equipment":ti,ab,kw OR "Apparatus and Instruments":ti,ab,kw OR "Instruments and Apparatus":ti,ab,kw OR "Supplies":ti,ab,kw OR "Inventories":ti,ab,kw OR "Inventory":ti,ab,kw OR "Medical Devices":ti,ab,kw OR "Medical Device":ti,ab,kw OR "Device, Medical":ti,ab,kw OR "Devices, Medical":ti,ab,kw OR "Devices":ti,ab,kw OR "Device":ti,ab,kw OR "Equipment":ti,ab,kw OR "bioinstrumentation":ti,ab,kw OR "biological instrumentation":ti,ab,kw OR "biomedical device":ti,ab,kw OR "biomedical device (physical object)":ti,ab,kw OR "biomedical equipment":ti,ab,kw OR "biomedical instrumentation":ti,ab,kw OR "clinical device":ti,ab,kw OR "clinical equipment":ti,ab,kw OR "durable medical equipment":ti,ab,kw OR "instrumentation, biological":ti,ab,kw OR "instrumentation, medical":ti,ab,kw OR "medical apparatus":ti,ab,kw OR "medical apparatus, equipment and supplies":ti,ab,kw OR "medical equipment":ti,ab,kw OR "medical instrument":ti,ab,kw OR "medical instrumentation":ti,ab,kw OR "medical supplies":ti,ab,kw OR "device-related":ti,ab,kw OR "medical device related":ti,ab,kw OR "appliances":ti,ab,kw OR "support surfaces":ti,ab,kw | 105,637 |
| **#16** | **#14 OR #15** | **144,658** |
| #17 | [mh "Pressure Ulcer"] | 817 |
| #18 | "Pressure Ulcer":ti,ab,kw OR "Pressure Ulcers":ti,ab,kw OR "Ulcer, Pressure":ti,ab,kw OR "Ulcers, Pressure":ti,ab,kw OR "Bedsore":ti,ab,kw OR "Bedsores":ti,ab,kw OR "Pressure Sore":ti,ab,kw OR "Pressure Sores":ti,ab,kw OR "Sore, Pressure":ti,ab,kw OR "Sores, Pressure":ti,ab,kw OR "Bed Sores":ti,ab,kw OR "Bed Sore":ti,ab,kw OR "Sore, Bed":ti,ab,kw OR "Sores, Bed":ti,ab,kw OR "Decubitus Ulcer":ti,ab,kw OR "Decubitus Ulcers":ti,ab,kw OR "Ulcer, Decubitus":ti,ab,kw OR "Ulcers, Decubitus":ti,ab,kw OR "decubital ulcer":ti,ab,kw OR "decubital ulcus":ti,ab,kw OR "decubitus ulceration":ti,ab,kw OR "decubitus ulcus":ti,ab,kw OR "decubus ulcer":ti,ab,kw OR "pressure injury":ti,ab,kw OR "ulcus decubitus":ti,ab,kw OR "pressure damage":ti,ab,kw | 2,151 |
| **#19** | **#17 OR #18** | **2,151** |
| #20 | "medical device related pressure ulcer":ti,ab,kw OR "device related pressure injury":ti,ab,kw OR "device related pressure ulcer":ti,ab,kw OR "MDRPI":ti,ab,kw OR "MDRPU":ti,ab,kw OR "medical device related pressure injury":ti,ab,kw OR "Medical Device-Related Pressure Injuries":ti,ab,kw | 10 |
| **#21** | **(#16 AND #19) OR #20** | **622** |
| **#22** | **#13 AND #21** | **164** |
| #23 | "prevention and control":ti,ab,kw | 4,434 |
| #24 | "prophylaxis":ti,ab,kw OR "preventive therapy":ti,ab,kw OR "prevention and control":ti,ab,kw OR "preventive measures":ti,ab,kw OR "prevention":ti,ab,kw OR "control":ti,ab,kw | 605,514 |
| #25 | [mh "Clinical Protocols"] | 20,013 |
| #26 | "Clinical Protocols":ti,ab,kw OR "Protocol, Clinical":ti,ab,kw OR "Protocols, Clinical":ti,ab,kw OR "Clinical Protocol":ti,ab,kw OR "Treatment Protocols":ti,ab,kw OR "Protocols, Treatment":ti,ab,kw OR "Treatment Protocol":ti,ab,kw OR "Clinical Research Protocol":ti,ab,kw OR "Research Protocols, Clinical":ti,ab,kw OR "Protocols, Clinical Research":ti,ab,kw OR "Research Protocol, Clinical":ti,ab,kw OR "Clinical Research Protocols":ti,ab,kw OR "Protocol, Clinical Research":ti,ab,kw OR "protocol":ti,ab,kw OR "protocols":ti,ab,kw | 137,738 |
| #27 | [mh "Patient Care Bundles"] | 41 |
| #28 | "Patient Care Bundles":ti,ab,kw OR "Bundle, Patient Care":ti,ab,kw OR "Bundles, Patient Care":ti,ab,kw OR "Care Bundle, Patient":ti,ab,kw OR "Care Bundles, Patient":ti,ab,kw OR "Patient Care Bundle":ti,ab,kw OR "Care Bundles":ti,ab,kw OR "Bundle, Care":ti,ab,kw OR "Bundles, Care":ti,ab,kw OR "Care Bundle":ti,ab,kw OR "healthcare bundle":ti,ab,kw OR "Bundle":ti,ab,kw OR "Bundles":ti,ab,kw | 2,488 |
| #29 | [mh "Algorithms"] | 4,791 |
| #30 | "Algorithms":ti,ab,kw OR "Algorithm":ti,ab,kw OR "algorhythm":ti,ab,kw OR "algorism":ti,ab,kw | 15,488 |
| **#31** | **{OR #23-#30}** | **698,986** |
| **#32** | **#22 AND #31** | **138** |

4)CINAHL

| S1 | (MH "Critical Care+") | 31,845 |
| --- | --- | --- |
| S2 | TI("Critical Care" OR "Care, Critical" OR "Intensive Care" OR "Care, Intensive" OR "Surgical Intensive Care" OR "Care, Surgical Intensive" OR "Intensive Care, Surgical" OR "intensive therapy" OR "therapy, intensive") OR AB("Critical Care" OR "Care, Critical" OR "Intensive Care" OR "Care, Intensive" OR "Surgical Intensive Care" OR "Care, Surgical Intensive" OR "Intensive Care, Surgical" OR "intensive therapy" OR "therapy, intensive") OR SU("Critical Care" OR "Care, Critical" OR "Intensive Care" OR "Care, Intensive" OR "Surgical Intensive Care" OR "Care, Surgical Intensive" OR "Intensive Care, Surgical" OR "intensive therapy" OR "therapy, intensive") | 144,597 |
| S3 | (MH "Critical Illness") | 14,658 |
| S4 | TI("Critical Illness" OR "Critical Illnesses" OR "Illness, Critical" OR "Illnesses, Critical" OR "Critically Ill" OR "serious illness" OR "seriously ill") OR AB("Critical Illness" OR "Critical Illnesses" OR "Illness, Critical" OR "Illnesses, Critical" OR "Critically Ill" OR "serious illness" OR "seriously ill") OR SU("Critical Illness" OR "Critical Illnesses" OR "Illness, Critical" OR "Illnesses, Critical" OR "Critically Ill" OR "serious illness" OR "seriously ill") | 45,074 |
| S5 | (MH "Intensive Care Units+") | 69,574 |
| S6 | TI("Intensive Care Units" OR "Intensive Care Unit" OR "Unit, Intensive Care" OR "ICU Intensive Care Units" OR "close attention unit" OR "combined medical and surgical ICU" OR "combined surgical and medical ICU" OR "critical care unit" OR "general ICU" OR "GICU" OR "GICUs" OR "ICU's" OR "intensive care department" OR "intensive therapy unit" OR "intensive treatment unit" OR "medical-surgery ICU" OR "medical/surgical ICU" OR "medical/surgical ICUs" OR "medico-surgical ICU" OR "mixed medical and surgical ICU" OR "mixed surgical and medical ICU" OR "respiratory care unit" OR "respiratory care units" OR "special care unit" OR "surgery/medical ICU" OR "surgical-medical ICUs" OR "surgical/medical ICU" OR "ICU" OR "SICU" OR "ITU" OR "CCU" OR "CTU" OR "HDU") OR AB("Intensive Care Units" OR "Intensive Care Unit" OR "Unit, Intensive Care" OR "ICU Intensive Care Units" OR "close attention unit" OR "combined medical and surgical ICU" OR "combined surgical and medical ICU" OR "critical care unit" OR "general ICU" OR "GICU" OR "GICUs" OR "ICU's" OR "intensive care department" OR "intensive therapy unit" OR "intensive treatment unit" OR "medical-surgery ICU" OR "medical/surgical ICU" OR "medical/surgical ICUs" OR "medico-surgical ICU" OR "mixed medical and surgical ICU" OR "mixed surgical and medical ICU" OR "respiratory care unit" OR "respiratory care units" OR "special care unit" OR "surgery/medical ICU" OR "surgical-medical ICUs" OR "surgical/medical ICU" OR "ICU" OR "SICU" OR "ITU" OR "CCU" OR "CTU" OR "HDU") OR SU("Intensive Care Units" OR "Intensive Care Unit" OR "Unit, Intensive Care" OR "ICU Intensive Care Units" OR "close attention unit" OR "combined medical and surgical ICU" OR "combined surgical and medical ICU" OR "critical care unit" OR "general ICU" OR "GICU" OR "GICUs" OR "ICU's" OR "intensive care department" OR "intensive therapy unit" OR "intensive treatment unit" OR "medical-surgery ICU" OR "medical/surgical ICU" OR "medical/surgical ICUs" OR "medico-surgical ICU" OR "mixed medical and surgical ICU" OR "mixed surgical and medical ICU" OR "respiratory care unit" OR "respiratory care units" OR "special care unit" OR "surgery/medical ICU" OR "surgical-medical ICUs" OR "surgical/medical ICU" OR "ICU" OR "SICU" OR "ITU" OR "CCU" OR "CTU" OR "HDU") | 104,728 |
| S7 | TI("intensive care medicine" OR "critical care medicine") OR AB("intensive care medicine" OR "critical care medicine") OR SU("intensive care medicine" OR "critical care medicine") | 2,733 |
| S8 | (MH "Hospitalization+") | 115,607 |
| S9 | TI("Hospitalization" OR "Hospitalizations" OR "hospital stay" OR "short stay hospitalization") OR AB("Hospitalization" OR "Hospitalizations" OR "hospital stay" OR "short stay hospitalization") OR SU("Hospitalization" OR "Hospitalizations" OR "hospital stay" OR "short stay hospitalization") | 113,543 |
| S10 | (MH "Life Support Care+") | 4,638 |
| S11 | TI("Life Support Care" OR "Care, Life Support" OR "Prolongation of Life" OR "Life Prolongation" OR "Extraordinary Treatment" OR "Extraordinary Treatments" OR "Treatment, Extraordinary" OR "Treatments, Extraordinary" OR "chronic treatment" OR "long term medical care" OR "long term therapy" OR "long term treatment" OR "long-term care" OR "medical care, long term" OR "treatment, long term" OR "coronary care" OR "cardiothoracic unit") OR AB("Life Support Care" OR "Care, Life Support" OR "Prolongation of Life" OR "Life Prolongation" OR "Extraordinary Treatment" OR "Extraordinary Treatments" OR "Treatment, Extraordinary" OR "Treatments, Extraordinary" OR "chronic treatment" OR "long term medical care" OR "long term therapy" OR "long term treatment" OR "long-term care" OR "medical care, long term" OR "treatment, long term" OR "coronary care" OR "cardiothoracic unit") OR SU("Life Support Care" OR "Care, Life Support" OR "Prolongation of Life" OR "Life Prolongation" OR "Extraordinary Treatment" OR "Extraordinary Treatments" OR "Treatment, Extraordinary" OR "Treatments, Extraordinary" OR "chronic treatment" OR "long term medical care" OR "long term therapy" OR "long term treatment" OR "long-term care" OR "medical care, long term" OR "treatment, long term" OR "coronary care" OR "cardiothoracic unit") | 50,051 |
| S12 | TI("critical patients" OR "critically ill patients") OR AB("critical patients" OR "critically ill patients") OR SU("critical patients" OR "critically ill patients") | 25,985 |
| **S13** | **S1 OR S2 OR S3 OR S4 OR S5 OR S6 OR S7 OR S8 OR S9 OR S10 OR S11 OR S12** | **383,478** |
| S14 | (MH "Equipment and Supplies+") | 490,618 |
| S15 | TI("Equipment and Supplies" OR "Supplies and Equipment" OR "Apparatus and Instruments" OR "Instruments and Apparatus" OR "Supplies" OR "Inventories" OR "Inventory" OR "Medical Devices" OR "Medical Device" OR "Device, Medical" OR "Devices, Medical" OR "Devices" OR "Device" OR "Equipment" OR "bioinstrumentation" OR "biological instrumentation" OR "biomedical device" OR "biomedical device (physical object)" OR "biomedical equipment" OR "biomedical instrumentation" OR "clinical device" OR "clinical equipment" OR "durable medical equipment" OR "instrumentation, biological" OR "instrumentation, medical" OR "medical apparatus" OR "medical apparatus, equipment and supplies" OR "medical equipment" OR "medical instrument" OR "medical instrumentation" OR "medical supplies" OR "device-related" OR "medical device related" OR "appliances" OR "support surfaces") OR AB("Equipment and Supplies" OR "Supplies and Equipment" OR "Apparatus and Instruments" OR "Instruments and Apparatus" OR "Supplies" OR "Inventories" OR "Inventory" OR "Medical Devices" OR "Medical Device" OR "Device, Medical" OR "Devices, Medical" OR "Devices" OR "Device" OR "Equipment" OR "bioinstrumentation" OR "biological instrumentation" OR "biomedical device" OR "biomedical device (physical object)" OR "biomedical equipment" OR "biomedical instrumentation" OR "clinical device" OR "clinical equipment" OR "durable medical equipment" OR "instrumentation, biological" OR "instrumentation, medical" OR "medical apparatus" OR "medical apparatus, equipment and supplies" OR "medical equipment" OR "medical instrument" OR "medical instrumentation" OR "medical supplies" OR "device-related" OR "medical device related" OR "appliances" OR "support surfaces") OR SU("Equipment and Supplies" OR "Supplies and Equipment" OR "Apparatus and Instruments" OR "Instruments and Apparatus" OR "Supplies" OR "Inventories" OR "Inventory" OR "Medical Devices" OR "Medical Device" OR "Device, Medical" OR "Devices, Medical" OR "Devices" OR "Device" OR "Equipment" OR "bioinstrumentation" OR "biological instrumentation" OR "biomedical device" OR "biomedical device (physical object)" OR "biomedical equipment" OR "biomedical instrumentation" OR "clinical device" OR "clinical equipment" OR "durable medical equipment" OR "instrumentation, biological" OR "instrumentation, medical" OR "medical apparatus" OR "medical apparatus, equipment and supplies" OR "medical equipment" OR "medical instrument" OR "medical instrumentation" OR "medical supplies" OR "device-related" OR "medical device related" OR "appliances" OR "support surfaces") | 404,743 |
| **S16** | **S14 OR S15** | **683,412** |
| S17 | (MH "Pressure Ulcer+") | 15,557 |
| S18 | TI("Pressure Ulcer" OR "Pressure Ulcers" OR "Ulcer, Pressure" OR "Ulcers, Pressure" OR "Bedsore" OR "Bedsores" OR "Pressure Sore" OR "Pressure Sores" OR "Sore, Pressure" OR "Sores, Pressure" OR "Bed Sores" OR "Bed Sore" OR "Sore, Bed" OR "Sores, Bed" OR "Decubitus Ulcer" OR "Decubitus Ulcers" OR "Ulcer, Decubitus" OR "Ulcers, Decubitus" OR "decubital ulcer" OR "decubital ulcus" OR "decubitus ulceration" OR "decubitus ulcus" OR "decubus ulcer" OR "pressure injury" OR "ulcus decubitus" OR "pressure damage") OR AB("Pressure Ulcer" OR "Pressure Ulcers" OR "Ulcer, Pressure" OR "Ulcers, Pressure" OR "Bedsore" OR "Bedsores" OR "Pressure Sore" OR "Pressure Sores" OR "Sore, Pressure" OR "Sores, Pressure" OR "Bed Sores" OR "Bed Sore" OR "Sore, Bed" OR "Sores, Bed" OR "Decubitus Ulcer" OR "Decubitus Ulcers" OR "Ulcer, Decubitus" OR "Ulcers, Decubitus" OR "decubital ulcer" OR "decubital ulcus" OR "decubitus ulceration" OR "decubitus ulcus" OR "decubus ulcer" OR "pressure injury" OR "ulcus decubitus" OR "pressure damage") OR SU("Pressure Ulcer" OR "Pressure Ulcers" OR "Ulcer, Pressure" OR "Ulcers, Pressure" OR "Bedsore" OR "Bedsores" OR "Pressure Sore" OR "Pressure Sores" OR "Sore, Pressure" OR "Sores, Pressure" OR "Bed Sores" OR "Bed Sore" OR "Sore, Bed" OR "Sores, Bed" OR "Decubitus Ulcer" OR "Decubitus Ulcers" OR "Ulcer, Decubitus" OR "Ulcers, Decubitus" OR "decubital ulcer" OR "decubital ulcus" OR "decubitus ulceration" OR "decubitus ulcus" OR "decubus ulcer" OR "pressure injury" OR "ulcus decubitus" OR "pressure damage") | 18,108 |
| **S19** | **S17 OR S18** | **18,208** |
| S20 | TI("medical device related pressure ulcer" OR "device related pressure injury" OR "device related pressure ulcer" OR "MDRPI" OR "MDRPU" OR "medical device related pressure injury" OR "Medical Device-Related Pressure Injuries") OR AB("medical device related pressure ulcer" OR "device related pressure injury" OR "device related pressure ulcer" OR "MDRPI" OR "MDRPU" OR "medical device related pressure injury" OR "Medical Device-Related Pressure Injuries") OR SU("medical device related pressure ulcer" OR "device related pressure injury" OR "device related pressure ulcer" OR "MDRPI" OR "MDRPU" OR "medical device related pressure injury" OR "Medical Device-Related Pressure Injuries") | 103 |
| **S21** | **(S16 AND S19) OR S20** | **5,555** |
| **S22** | **S13 AND S21** | **976** |
| S23 | TI("prevention and control") OR AB("prevention and control") OR SU("prevention and control") | 636,683 |
| S24 | TI("prophylaxis" OR "preventive therapy" OR "prevention and control" OR "preventive measures" OR "prevention" OR "control") OR AB("prophylaxis" OR "preventive therapy" OR "prevention and control" OR "preventive measures" OR "prevention" OR "control") OR SU("prophylaxis" OR "preventive therapy" OR "prevention and control" OR "preventive measures" OR "prevention" OR "control") | 1,264,507 |
| S25 | TI("Clinical Protocols" OR "Protocol, Clinical" OR "Protocols, Clinical" OR "Clinical Protocol" OR "Treatment Protocols" OR "Protocols, Treatment" OR "Treatment Protocol" OR "Clinical Research Protocol" OR "Research Protocols, Clinical" OR "Protocols, Clinical Research" OR "Research Protocol, Clinical" OR "Clinical Research Protocols" OR "Protocol, Clinical Research" OR "protocol" OR "protocols") OR AB("Clinical Protocols" OR "Protocol, Clinical" OR "Protocols, Clinical" OR "Clinical Protocol" OR "Treatment Protocols" OR "Protocols, Treatment" OR "Treatment Protocol" OR "Clinical Research Protocol" OR "Research Protocols, Clinical" OR "Protocols, Clinical Research" OR "Research Protocol, Clinical" OR "Clinical Research Protocols" OR "Protocol, Clinical Research" OR "protocol" OR "protocols") OR SU("Clinical Protocols" OR "Protocol, Clinical" OR "Protocols, Clinical" OR "Clinical Protocol" OR "Treatment Protocols" OR "Protocols, Treatment" OR "Treatment Protocol" OR "Clinical Research Protocol" OR "Research Protocols, Clinical" OR "Protocols, Clinical Research" OR "Research Protocol, Clinical" OR "Clinical Research Protocols" OR "Protocol, Clinical Research" OR "protocol" OR "protocols") | 140,939 |
| S26 | TI("Patient Care Bundles" OR "Bundle, Patient Care" OR "Bundles, Patient Care" OR "Care Bundle, Patient" OR "Care Bundles, Patient" OR "Patient Care Bundle" OR "Care Bundles" OR "Bundle, Care" OR "Bundles, Care" OR "Care Bundle" OR "healthcare bundle" OR "Bundle" OR "Bundles") OR AB("Patient Care Bundles" OR "Bundle, Patient Care" OR "Bundles, Patient Care" OR "Care Bundle, Patient" OR "Care Bundles, Patient" OR "Patient Care Bundle" OR "Care Bundles" OR "Bundle, Care" OR "Bundles, Care" OR "Care Bundle" OR "healthcare bundle" OR "Bundle" OR "Bundles") OR SU("Patient Care Bundles" OR "Bundle, Patient Care" OR "Bundles, Patient Care" OR "Care Bundle, Patient" OR "Care Bundles, Patient" OR "Patient Care Bundle" OR "Care Bundles" OR "Bundle, Care" OR "Bundles, Care" OR "Care Bundle" OR "healthcare bundle" OR "Bundle" OR "Bundles") | 12,678 |
| S27 | (MH "Algorithms") | 43,393 |
| S28 | TI("Algorithms" OR "Algorithm" OR "algorhythm" OR "algorism") OR AB("Algorithms" OR "Algorithm" OR "algorhythm" OR "algorism") OR SU("Algorithms" OR "Algorithm" OR "algorhythm" OR "algorism") | 68,319 |
| **S29** | **S23 OR S24 OR S25 OR S26 OR S27 OR S28** | **1,428,590** |
| **S30** | **S22 AND S29** | **728** |
